# Spontaneous Pneumomediastinum Presenting as Dysphonia

**DOI:** 10.7759/cureus.100250

**Published:** 2025-12-28

**Authors:** Jacob Weaver, Rachel Cox, Eric Karr

**Affiliations:** 1 Internal Medicine, Wright State University, Dayton, USA; 2 Internal Medicine, Wright Patterson Medical Center, Wright-Patterson Air Force Base, USA

**Keywords:** chest pain, computed tomography (ct), dysphonia workup, dyspnea, esophagram, hamman’s syndrome, macklin effect, pneumomediastinum (pm), spontaneous pneumomediastinum (spm), subcutaneous emphysema

## Abstract

Spontaneous pneumomediastinum is a rare condition describing air within the mediastinum from a non-traumatic etiology from an intrathoracic or extrathoracic source. It typically affects young men through a mechanism known as the Macklin effect, in which alveolar air travels along bronchovascular sheaths into the mediastinum. There are known predisposing conditions as well as activities that can precipitate spontaneous pneumomediastinum, which is generally self-limited, but in rare circumstances, can lead to serious complications such as tension pneumomediastinum, mediastinitis, and tension pneumothorax. The classic triad of symptoms for this condition includes chest pain, subcutaneous emphysema, and dyspnea. We present a case of spontaneous pneumomediastinum in a 19-year-old male patient with the chief complaint of dysphonia after repeated heavy lifting three days prior to presentation.

Spontaneous mediastinum after heavy lifting has been reported previously; however, our case is unique in that the patient presented with dysphonia without chest pain or dyspnea. This condition is important to keep in mind as spontaneous pneumomediastinum is typically self-limiting and can be managed conservatively, reducing unnecessary resource utilization. A chest radiograph is the diagnostic test of choice, and further diagnostic studies may not be necessary. In our case, aerodigestive injury was ruled out with a swallow study because there was a history of occasional vomiting and an unknown source of pneumomediastinum. Although spontaneous pneumomediastinum more commonly presents with chest pain and dyspnea, it is an overall rare condition and should be considered in cases of dysphonia as well, as in this case. Prompt diagnosis of spontaneous pneumomediastinum is important to both monitor for complications and avoid overtreatment.

## Introduction

Pneumomediastinum refers to the presence of free air within the mediastinum, a condition first recognized in the late 19th century when limited understanding of thoracic disease made diagnosis rare. Despite advances in imaging and clinical knowledge, it remains underdiagnosed today. Early descriptions include Laennec's 1819 report of "interlobular emphysema" in the context of trauma, though an even earlier likely case was described by Simmons in 1783 involving postpartum subcutaneous emphysema. In 1939, Hamman characterized "spontaneous mediastinal emphysema" in otherwise healthy individuals without trauma or underlying disease, a distinction that led to the term "medical mediastinal emphysema", later abandoned in favor of "pneumomediastinum". Hamman's careful clinical descriptions ultimately popularized the term "spontaneous pneumomediastinum", which is often referred to as Hamman's syndrome in recognition of his contributions. The pathophysiologic basis of the condition was clarified in 1944 by Macklin and Macklin, who demonstrated that rupture of terminal alveoli allows air to dissect along perivascular sheaths from the pulmonary interstitium into the mediastinum, known as the Macklin effect [1,2].

Spontaneous or primary pneumomediastinum is a rare condition with a reported incidence of 1:7000-1:45000 hospital admissions, but is thought to be underestimated due to a lack of awareness leading to missed diagnosis [3]. Spontaneous pneumomediastinum is more common in males (3.6:1) [4] and generally appears in a younger population between 10 and 30 years old [3]. Pneumomediastinum can be either spontaneous (primary) or secondary in nature. Secondary pneumomediastinum usually arises from trauma or iatrogenic causes, whereas spontaneous pneumomediastinum is typically atraumatic and can have predisposing conditions and precipitating factors. Predisposing conditions create favorable conditions for spontaneous pneumomediastinum to occur, but do not directly cause a pneumomediastinum. Some of the common predisposing conditions include tobacco smoking, asthma [3], and, more recently, COVID-19 [5]. Precipitating factors are the events that trigger a spontaneous pneumomediastinum to occur and include emesis, coughing, asthma exacerbation, and physical exercise [3].

Spontaneous pneumomediastinum is generally benign and self-limiting but can lead to complications such as hypertensive pneumomediastinum, mediastinitis [3], and tension pneumothorax [6]. Patients who are at higher risk may include males with a tall and lean habitus and a history of predisposing conditions [4]. The classic symptoms in a patient presenting with pneumomediastinum include chest pain, subcutaneous emphysema, and dyspnea. Systolic crackles heard at the left sternal border describe Hamman's sign, which is pathognomonic for pneumomediastinum and may be described like balloons rubbing together [3]. Diagnosis is made using chest X-ray followed by a chest computed tomography if the X-ray is negative, but suspicion remains high or if there is concern for esophageal involvement. Supportive care is provided for uncomplicated pneumomediastinum and includes bed rest, oxygen therapy, and pain management [4]. In this case report, we present a case of spontaneous pneumomediastinum in a young male patient with the chief complaint of dysphonia after repeated heavy lifting.

## Case presentation

A 19-year-old male with no past medical history presented to the emergency department with two days of voice changes and neck pain. The patient reported that his voice sounded more nasally than his baseline. Three days prior to presentation, the patient had been working a new job, lifting fifty-pound barrels over his head repeatedly for about five hours. He first noticed the neck pain and voice change on the following day. He denied any prior similar episodes. The neck pain had resolved on the day of his visit, but his voice changes persisted, prompting his visit. He denied shortness of breath, chest pain, cough, sore throat, fever, or chills. He denied a recent skin or soft tissue injury. The patient did report occasional episodes of vomiting prior to the onset of his voice changes. He had no history of recent illnesses or traumatic injury, and he did not smoke or vape. He also denied drug and alcohol use. He did not have any pertinent surgical history. 

In the emergency department, the patient was afebrile, tachycardic (HR of 109 bpm), and hypertensive (157/83 mmHg) likely due to a stress reaction, with a normal respiratory rate and 100% oxygen saturation on room air. The physical exam was pertinent for a nasal voice different from the patient's baseline, as well as crepitus of the neck with extension into the chest. The patient otherwise had clear lungs and was not in acute distress. His skin was warm, dry, and intact throughout. Cardiac examination revealed tachycardia with a regular rhythm and no murmurs, rubs, or gallops. No abnormalities noted on oral exam. Laboratory tests (Tables 1-2) were unremarkable; however, a slightly elevated anion gap could be due to starvation ketosis in the setting of a remote history of vomiting, although this was not confirmed. Additionally, an anion gap of 16 is considered normal at other institutions. In the absence of fever or leukocytosis, C-reactive protein and procalcitonin were not indicated. The basic laboratory work performed in the emergency room is presented to demonstrate that another acute process was unlikely, and the lab work did not influence further management. 

**Table 1:** Complete blood count collected in the emergency room

Complete blood count	Value	Reference
White blood cell count	8.2 ×10³/µL	3.5-10.9 ×10³/µL
Red blood cells	4.64 ×10^6^/µL	4.14-5.80 ×10^6^/µL
Hemoglobin	13.8 g/dL	13.0-17.7 g/dL
Hematocrit	41.20%	37.5-51.0%
Platelets	270 ×10³/µL	140-400 ×10³/µL

**Table 2:** Basic metabolic panel collected in the emergency room

Basic metabolic panel	Value	Reference
Sodium	138 mEq/L	135-148 mEq/L
Potassium	3.5 mEq/L	3.4-5.3 mEq/L
Chloride	103 mEq/L	96-110 mEq/L
Carbon dioxide	19 mEq/L	19-32 mEq/L
Blood urea nitrogen	16 mg/dL	3-29 mg/dL
Creatinine	1.0 mg/dL	0.4-1.4 mg/dL
Glucose	82 mg/dL	70-99 mg/dL
Calcium	8.6 mg/dL	8.5-10.5 mg/dL
Anion gap	16	5-15

**Figure 1:**
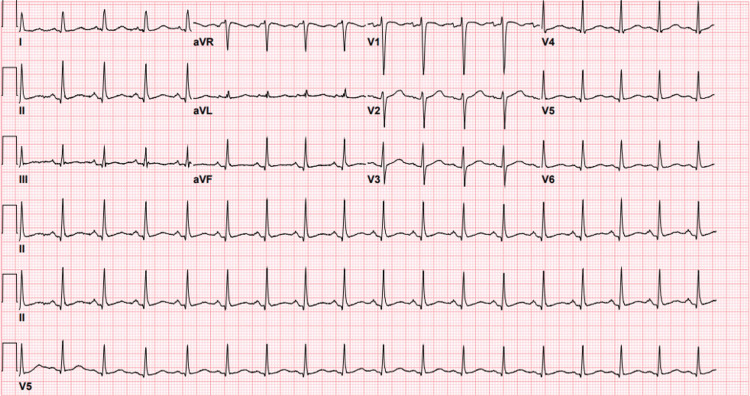
12-lead electrocardiogram (ECG) showed normal sinus tachycardia

**Figure 2:**
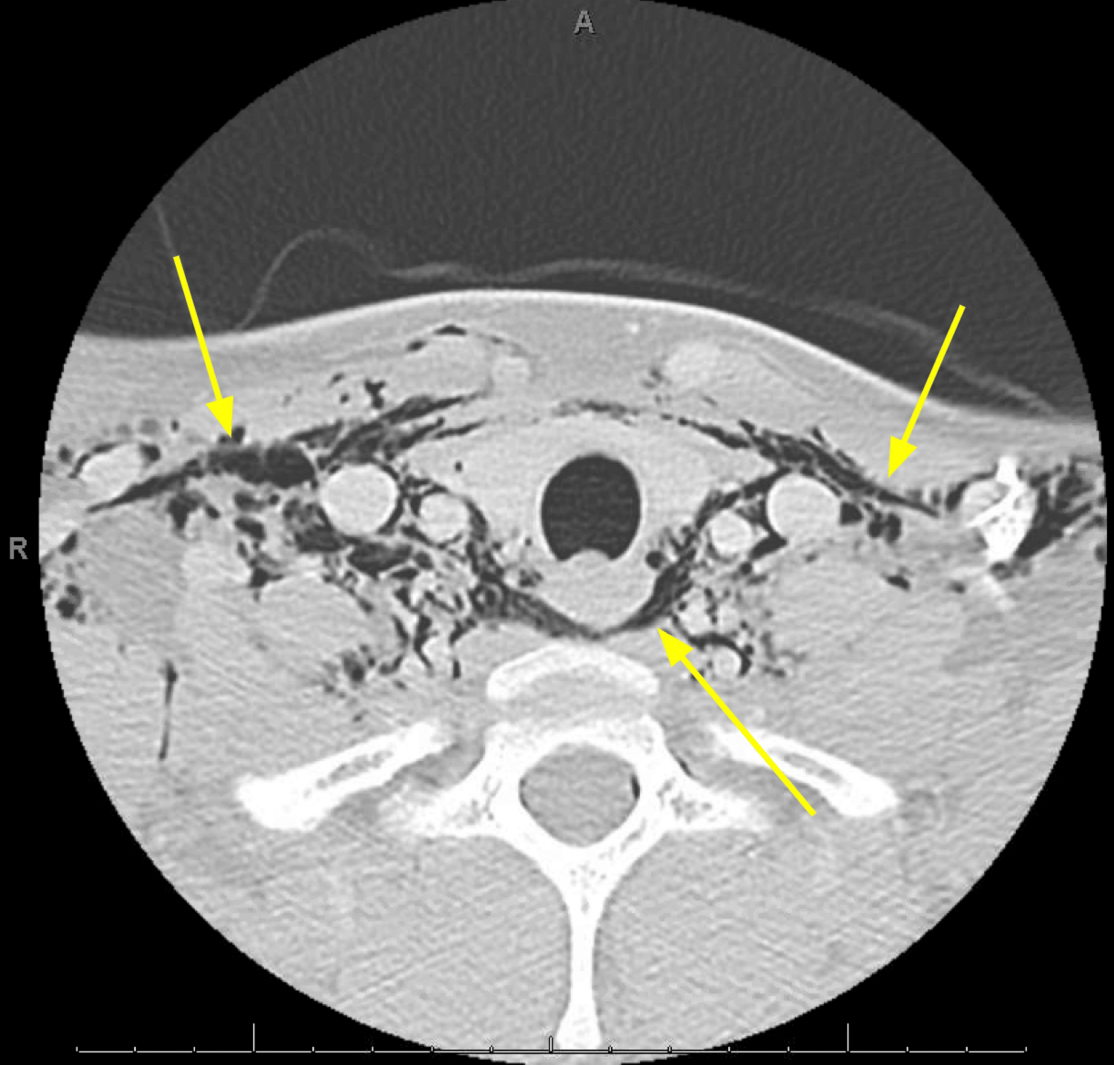
Axial computed tomography with contrast of the neck and soft tissues at the thoracic inlet showing subcutaneous emphysema within the neck

**Figure 3:**
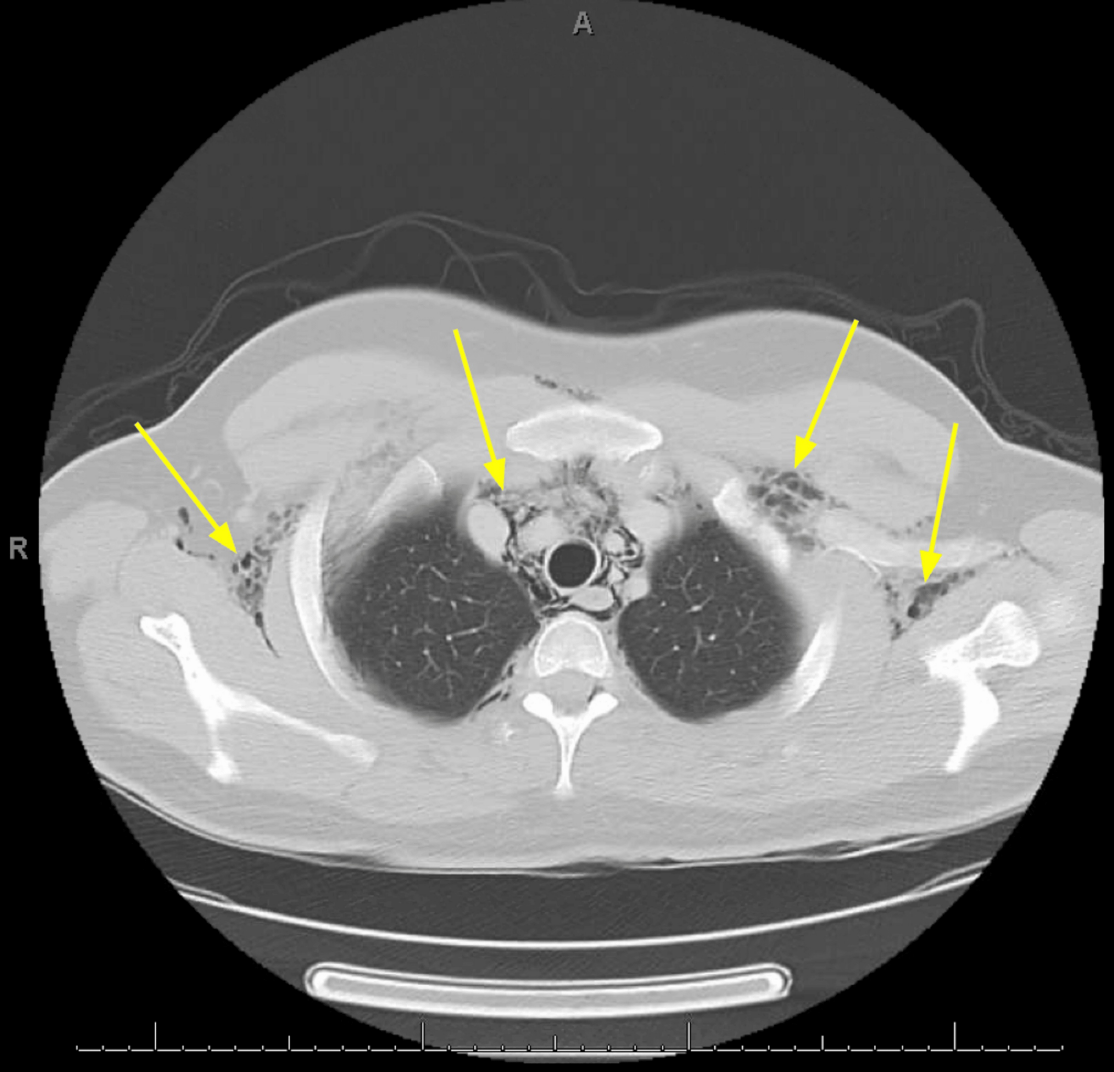
Computed tomography with contrast of the chest at the level of the superior mediastinum showing subcutaneous emphysema within the upper chest

Twelve-lead electrocardiogram revealed sinus tachycardia without significant arrhythmia or ischemic changes. Computed tomography (CT) of the neck soft tissue with contrast (Figure 1) and CT of the chest with contrast (Figures 2-3) was obtained to further characterize the crepitus noted on exam, which revealed extensive air in soft tissue of the neck, upper chest wall, and upper mediastinum. Cardiothoracic surgery was consulted by the emergency department, who recommended obtaining an esophagram to assess for esophageal injury due to the history of recent vomiting. The esophagram was obtained and did not reveal esophageal injury. The patient was admitted overnight for observation, during which he remained hemodynamically stable and did not develop new or worsening symptoms. He was subsequently discharged home the following day in stable condition. He was given strict return precautions and educated on his condition.

**Figure 4:**
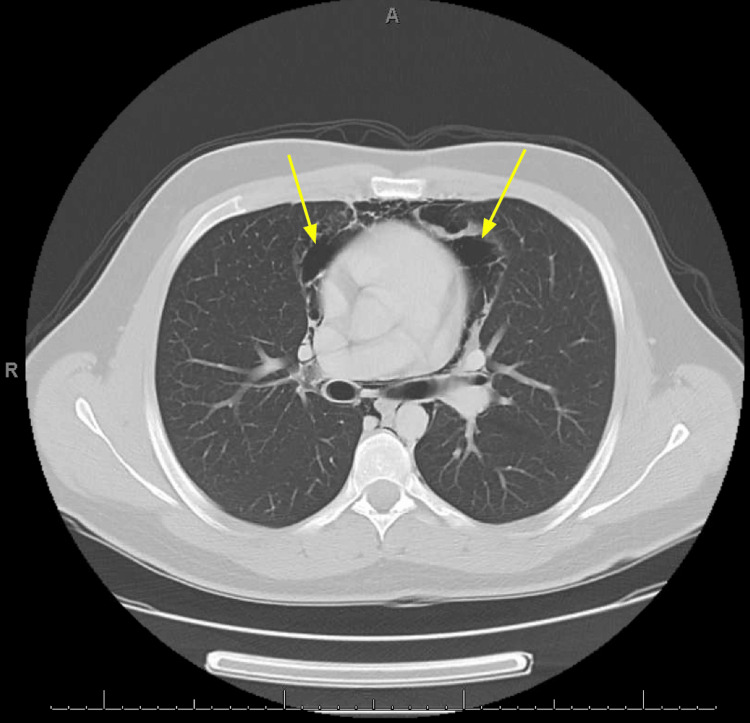
Computed tomography with contrast of the chest at the level of the inferior mediastinum showing air within the mediastinum

## Discussion

This case report presents a case of spontaneous pneumomediastinum, a rare, non-traumatic cause of subcutaneous emphysema in a young male patient after repeated heavy lifting. Spontaneous mediastinum after heavy lifting has been reported previously [6]; however, our case is unique in that the patient presented with dysphonia without chest pain or dyspnea. Although spontaneous pneumomediastinum more commonly presents with chest pain and dyspnea [5], it is an overall rare condition and should be considered in cases of dysphonia as well, as in this case. In a recent systematic review, dysphonia was the presenting chief complaint in 2% of cases out of 1134 cases [5]. It is suspected that the pathophysiological mechanism of dysphonia is due to compression of the recurrent laryngeal nerve [7]. 

There is a gap in the literature addressing the workup and management of pneumomediastinum due to the wide variety of etiologies that can precipitate the condition [4]. However, recent literature suggests that pneumomediastinum should be evaluated based on whether the patient is at high or low risk for esophageal involvement. In this algorithm, history is crucial, as a patient with a significant cough would be in the low-risk category and a patient with significant vomiting would be in the high-risk category. Both approaches are very similar except that there is a more aggressive evaluation for complications and esophageal etiologies in the high-risk category [4].

In this case, the patient presented with a history of vomiting, placing the patient in the category of higher risk for esophageal involvement. Therefore, CT was used to evaluate the patient for pneumomediastinum, and aerodigestive injury was ruled out with a swallow study. The patient did not have signs of esophageal injury or other complications, such as pneumothorax, and was treated with predominantly supportive care. Supportive care includes bed rest, analgesia, and oxygen therapy, which is thought to increase the diffusion pressure of nitrogen, leading to promotion of free air absorption in the mediastinum [5]. Like the suggested management of pneumomediastinum in the literature, the patient was discharged after a brief observation period due to their afebrile state and unremarkable labs, making mediastinitis a less likely complication, and their lack of findings of other complications, such as pneumothorax.

Prompt diagnosis of spontaneous pneumomediastinum is important to both monitor for complications and avoid overtreatment. The differential diagnosis generally includes conditions such as acute coronary syndrome, pericarditis, pneumothorax, pulmonary embolism, tracheobronchial tree rupture, and Boerhaave's syndrome [3]. Spontaneous pneumomediastinum is important to keep in mind, as although it has the potential to progress to more fatal disease states, such as mediastinitis, spontaneous pneumomediastinum is typically self-limiting and can be managed conservatively. If complications are found, they should be treated accordingly. If the patient presents with evidence of infection or concern for esophageal perforation, prophylactic antibiotics may be considered to prevent mediastinitis. If the patient is clinically stable with an uncomplicated pneumomediastinum, early discharge may be considered [4].

## Conclusions

Pneumomediastinum is a rare and likely underdiagnosed condition that is typically benign but may lead to serious complications. A chief complaint of dysphonia secondary to pneumomediastinum is even more rare, especially in the absence of chest pain or dyspnea. In conclusion, despite its low frequency, spontaneous pneumomediastinum should be considered in the differential diagnosis of acute chest pain because it requires a high index of suspicion and because the treatment protocol differs substantially from that of many other processes with similar clinical features. Delayed diagnosis of an uncomplicated spontaneous pneumomediastinum can lead to unwarranted long admissions, unnecessary tests, and treatments in a young patient population that may be more susceptible to adverse effects. In most cases, diagnosis can be established with chest radiography, and management is conservative, consisting of observation, rest, and supplemental oxygen, with invasive interventions reserved for patients with suspected complications or specific underlying etiologies. Therefore, recognizing pneumomediastinum early is crucial to preventing further harm to patients by being aware of the possible complications and reducing exposure to potential iatrogenic injuries and sequelae.

## References

[1] Campbell-Silva S, Campbell-Quintero S, Díaz-Rodríguez DC, Campbell-Quintero S, Castro-González I (2025). Spontaneous pneumomediastinum: a narrative review offering a new perspective on its definition and classification. Cureus.

[2] Murayama S, Gibo S (2014). Spontaneous pneumomediastinum and Macklin effect: overview and appearance on computed tomography. World J Radiol.

[3] Meireles J, Neves S, Castro A, França M (2011). Spontaneous pneumomediastinum revisited. Respir Med CME.

[4] Susai CJ, Banks KC, Alcasid NJ, Velotta JB (2024). A clinical review of spontaneous pneumomediastinum. Mediastinum.

[5] Talwar A, Rajeev A, Rachapudi S, Khan S, Singh V, Talwar A (2024). Spontaneous pneumomediastinum: a comprehensive review of diagnosis and management. Intractable Rare Dis Res.

[6] Swaminathan N, Agrawal A, Ram P (2019). Pneumomediastinum in a heavy weightlifter. Eur J Case Rep Intern Med.

[7] North L, Sulman C (2019). Subcutaneous emphysema and vocal fold paresis as a complication of a dental procedure. Int J Pediatr Otorhinolaryngol.

